# A Case of Post-Partum Hemorrhage Treated by Hyatt’s Method

**Published:** 1878-04-20

**Authors:** T. A. Woodley

**Affiliations:** Kinston, N. C.


### A CASE OF POST-PARTUM HEMORRHAGE TREATED BY HYATT'S METHOD.

I was called on the night of October 20th, 1877, to visit Mrs. M. T., age 22 years, in labor with her first child. I arrived at 2 o'clock a. m., and was informed that she had been in labor since 4 o'clock of yesterday afternoon. At the time of my arrival the bag of waters had been ruptured, and the pains were recurring every twenty or thirty minutes. I made a digital examination, and found the head presenting the left occipito anterior; os fully dilated, and the conjugate diameter less than four inches. I waited until morning, entertaining the vain hope that nature would be sufficient to effect a delivery. I applied forceps, and after continued and repeated efforts was unable to effect delivery, owing to the large size of the child's head and smallness of the superior strait.

Finding it necessary to resort to craniotomy, and not having the necessary instruments with me, I sent for Dr. Hyatt to assist me. He came about 2½ o'clock on the 21st, and after examining the patient agreed with me as to the expediency of the proposed operation. In a short time we succeeded in delivering the head, allowing the body of the child to remain in the uterus and vagina. We were apprehensive of uterine inertia and post-partum hemorrhage; but we hoped that by waiting we would give the uterus sufficient time to regain power and remain contracted after it was emptied of its contents. After waiting about half an hour, we delivered the body of the child, and found the placenta attached to the fundus of the uterus, and hemorrhage began to flow profusely. While I was delivering the placenta, Dr. H. gave the patient a dose of fl. ex. of ergot.

The flooding continuing after the removal of the placenta and blood clots, Dr. Hyatt handed me a rubber bag, which I passed into the cavity of the uterus. After its introduction, I distended it with cold water by means of a Davidson syringe. I used about a pint and a half of water, which was sufficient to arrest the hemorrhage completely. The bag adapted itself to the open ends of the bleeding vessels and completely sealed them. While I held the bag in the uterus, Dr. Hyatt kneaded the uterus through the abdominal walls. In a few minutes, contraction of the organ came on and expelled the bag. There was no further trouble, and up to the present time the patient's convalescence has been in every way normal.

This method of arresting post-partum hemorrhage is certainly the speediest, safest and most effectual to which I have ever resorted. During the last twenty years, I have seen a number of cases of this alarming accident, and have used all the usual remedies recommended; but the method of treatment adopted in the case just reported, gives the best result of any known to me. I would suggest to those engaged in the practice of obstetrics to always be equipped with a number of rubber bags (Barnes' dilators will do) and a Davidson syringe. Then the doctor need not fear any case of post-partum hemorrhage which may be due to uterine inertia.—*T. A. Woodley, M. D., Kinston, N. C., in Virginia Medical Monthly.*

